# Genetic analysis of endangered hog deer (*Axis porcinus*) reveals two distinct lineages from the Indian subcontinent

**DOI:** 10.1038/s41598-018-34482-9

**Published:** 2018-11-05

**Authors:** Sandeep Kumar Gupta, Ajit Kumar, Sangeeta Angom, Bhim Singh, Mirza Ghazanfar Ullah Ghazi, Chongpi Tuboi, Syed Ainul Hussain

**Affiliations:** Wildlife Institute of India, P.O. Box # 18, Chandrabani, Dehra Dun, 248001 Uttarakhand India

## Abstract

The hog deer (*Axis porcinus*) is threatened by habitat alteration, fragmentation, and poaching, which have led to a drastic decline of its wild population. Two subspecies of *A. porcinus* have been described from its distribution range. *A. p. porcinus* is reported to occur from Pakistan along the Himalayan foothills through Nepal, India and Myanmar, and *A. p. annamiticus* is found in Thailand, Indo-China, Laos, Cambodia, and Vietnam. However, the current distribution range of *A. p. annamiticus* is still unclear. We used the partial control region (CR) of mitochondrial DNA (mtDNA) and seven microsatellite loci to investigate the intra-species structure, differentiation, and demographic history of hog deer populations from three landscapes, the Terai Arc, Northeast, and Indo-Burma (Keibul Lamjao National Park (KLNP), Manipur, India) landscapes. We also carried out divergence time estimation using the complete mitogenome. The level of variation was ~4%, and the time of divergence of the KLNP population and the other Indian populations was about 0.22 Mya, i.e., during the last glaciation periods of the Late Pleistocene/Early Holocene. The KLNP haplotypes of the control region were shared with the Southeast Asian subspecies, *A. p. annamiticus*. The results of the investigations of the microsatellite loci supported the mtDNA results unambiguously. Two genetically distinct lineages are found in India: one is found from the Terai Arc to Assam (*A. p. porcinus*) and the other in Manipur (*A. p. annamiticus*). The genetic diversity in KLNP was low and exhibited a higher degree of genetic differentiation compared with major Indian populations. The Bayesian skyline plots indicated that after a long phase of historic demographic stability, the populations of both the lineages of hog deer suffered pronounced declines during the period from ~800 years BP to 5000 years BP. In summary, our finding provided evidence that the KLNP population is probably a prime, isolated and sustaining stock of *A. p. annamiticus* and should be managed as evolutionarily significant units (ESUs).

## Introduction

The hog deer (*Axis porcinus*), is an endangered species in the IUCN Red List and is protected under Schedule I of the Indian Wild Life (Protection) Act, 1972. Historically, it was distributed from Pakistan, in the west, to non-Sundaic Southeast Asia^1–4^. It is a very primitive cervid, reported to have been present since the Pliocene and Pleistocene, when forests were interspersed with open areas across Europe and Asia^5^. At the beginning of the 20th century, it was widely distributed throughout the Southeast Asian countries. However, in recent decades, continued depletion of its habitat have resulted in a population decline, and it is confined to a few localities in its historical distribution range^4,6,7^. It is supposed to be extinct in 35 localities of Southeast Asia where it was found^4,6^. However, the population of hog deer in Kaziranga National Park (KZNP) is persistent^8^. Keibul Lamjao National Park (KLNP), Manipur is a part of the Indo-Burma biodiversity hotspot, which holds a small population of hog deer along with the sympatric Eld’s deer (*Rucervus eldii eldii*).

Two sub-species of the hog deer have been reported from its range. *A. p. porcinus* (western race) is distributed from Pakistan^9^ and the terai grasslands (along the Himalayan foothills, from Punjab, in the west, to Arunachal Pradesh, in the east) through Nepal to Myanmar and in the floodplains of the rivers Ganges and Brahmaputra^6,10^. It has been re-introduced in Thailand and is being managed in captivity and semi-wild conditions^4^. According to the IUCN Red List, *A. p. annamiticus* (eastern race) is found in Thailand, Indo-China, Laos, Cambodia, and Vietnam^11–14^. The historical distribution of the eastern race of the hog deer (*A. p. annamiticus*) is still unknown^4,15^. A small and fragmented population of *A. p. annamiticus* is known to be found in Cambodia. This is presumed to be its last known wild stock^4,16,17^. Due to the bleak distribution of *A. p. annamiticus*, the western limit of its range could not be identified properly. Such isolated and small populations are always of concern as they are prone to loss of genetic diversity, resulting in their inability to adapt to a changing environment^18,19^.

Several studies have been conducted on the genetic variations and phylogeny of cervids^20–25^. One phylogenetic study recommended a change in the taxonomy hog deer^21^; however, it was subsequently found that the hog deer sample used was a case of mistaken identity^23^. Hence, reliable samples are essential for resolving phylogenetic relationships. In spite of the vulnerability of the hog deer to changing environmental conditions and anthropogenic pressure, very few studies have been conducted on the systematics, phylogenetic status, and ecology of this species. It is believed that the genetic diversity of the hog deer population is eroding^24^. Overall, the population boundaries and the genetic structuring of the hog deer remain unclear, and the classification of *A. porcinus* subspecies is still under dispute. An appropriate conservation and management plan for an endangered species can be prepared by incorporating reliable knowledge about the existing genetic diversity and population structure from across the geographic range of the species. Due to the maternal mode of transmission, mitochondrial DNA (mtDNA) provides information about female-mediated gene flow, whereas microsatellite markers are widely used to investigate the genetic variation and genetic structuring of populations^25,26^. Investigation of the genetic pattern across the distribution range of this little studied species, the hog deer, will allow insights into its evolutionary relationships and enable better conservation planning. Herein, we aimed to assess the genetic structure and differentiation among the extant hog deer populations to answering the key question of whether all Indian populations of the hog deer belong to a single evolutionary unit. We used complete mitogenome sequence data to estimate the divergence time; the partial control region (CR) to assess the genetic diversity, demography, and phylogeographic patterns; and microsatellite loci to address the genetic variation, differentiation, and population genetic structure. On the basis of genetic data, we discuss the relevance of our results to the biogeographical changes that took place during the Late Pleistocene in Northeast India.

## Results

### mtDNA control region sequence polymorphism and haplotype diversity

**Figure 1:**
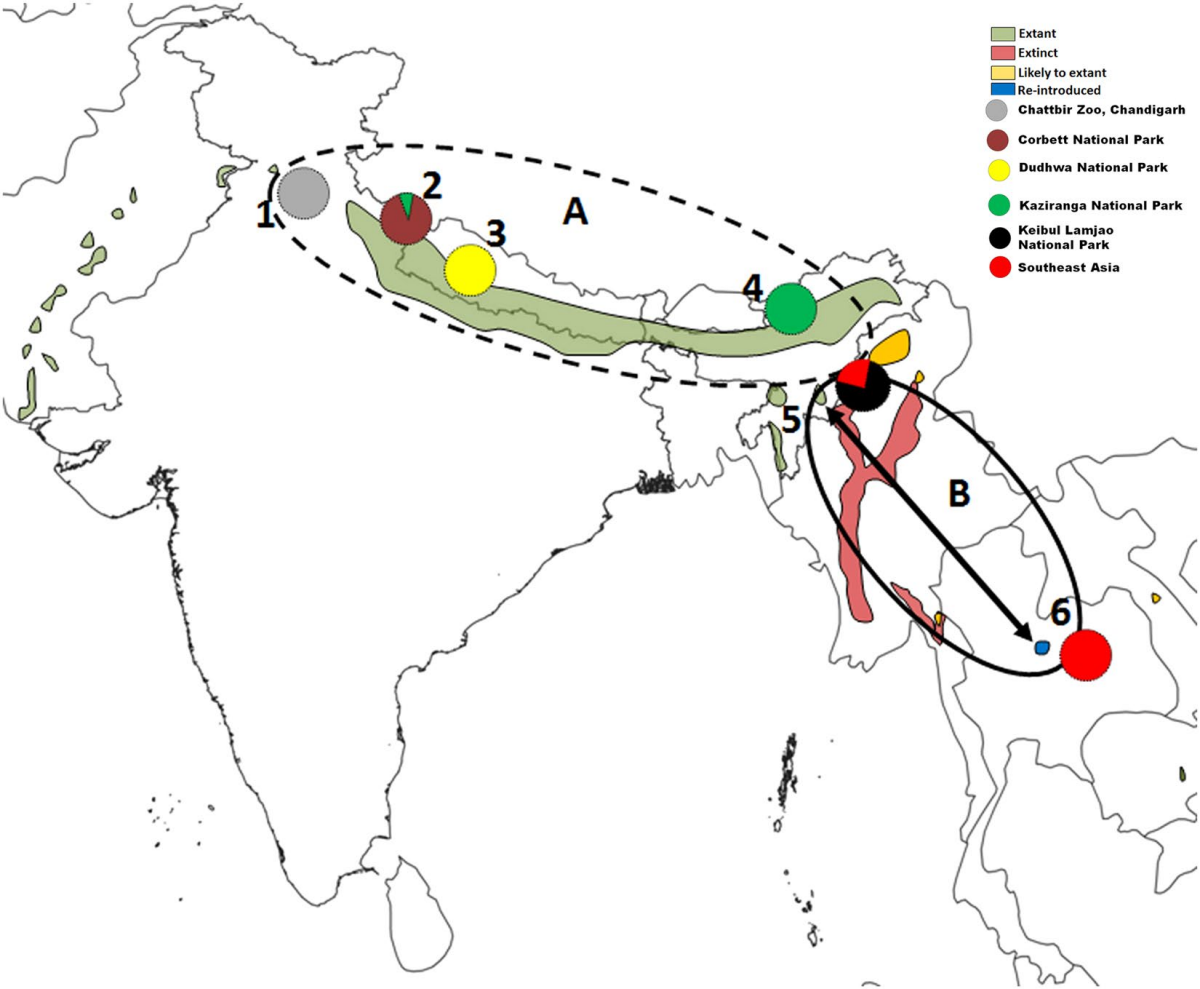
Mitochondrial DNA control region based haplotype sharing and the distribution map of hog deer (*Axis porcinus*) adapted from IUCN^4^ and created using the ArcGIS 10.2 software package. Dotted circle A and solid circle B represents two distinct lineages of *A. p. porcinus* and *A. p. annamiticus*, respectively.

To estimate the genetic diversity, we generated a 460 bp fragment of the CR from 77 samples. We compared the CR sequences of Indian origin with the 19 GenBank samples. Of the 77 in-house Indian sequences, 48 were of the KLNP population, and of the 19 GenBank sequences, 12 were of known *A. p. annamiticus*. We obtained 38 variable sites among the CR sequences. Of these, 14 polymorphic sites were singletons, and 24 were parsimony informative. These sequences were grouped into 26 haplotypes, and there were different numbers of individuals in each haplotype. Of these, 13 haplotypes (Hap 1 to Hap 13) were observed in Indian samples, and these were submitted to GenBank (MH392156 to MH392168). Haplotypes (Hap) 1 to 3 were observed in the samples from Corbett National Park (CNP), and Hap 1 was shared with Delhi Zoo (DZ). Haps 4 and 5 were found in Dudhwa National Park (DNP), and the unique Hap 6 was noted in the Chandigarh Zoo (CHZ) sample. Haps 7 to 10 were from KZNP, Assam, and Haps 11 to 13 were observed in KLNP, Manipur. Haps 12 to 26 were detected in the Southeast Asian hog deer (eastern races). Interestingly, Haps 12 and 13 were common in the KLNP population and Southeast Asian samples (Fig. 1).

When the haplotypes of the western and eastern lineages were compared, 10 haplotypes clustered in lineage 1 (DZ, CNP, DNP, CHZ, and KZNP), and 16 clustered in lineage 2 (KLNP and Southeast Asian origin). The haplotypes (h) and nucleotide diversity (π) of lineage 1 were 0.831 ± 0.043 and 0.031 ± 0.001, and those of lineage 2 were 0.66 ± 0.059 and 0.002 ± 0.0003. The overall mean intra-population genetic distance of lineage 1 (0.018 ± 0.004) was much greater than that of lineage 2 (0.004 ± 0.003). The genetic distance between the KLNP population and the rest of the Indian population was 0.028 ± 0.006. The average genetic distance between the groups indicated that the KLNP population was genetically closer to the eastern race, *A. p. annamiticus* (0.005 ± 0.001) than to the western race, *A. p. porcinus* (Table 1).

**Table 1:** Genetic differentiation among the hog deer populations. The pairwise *F*_*ST*_ values based on microsatellite loci are above the diagonal and genetic distances based on mtDNA control region (Tamura 3-Parameter Model (T92 + G) using a discrete Gamma distribution) are below the diagonal.

Populations	1	2	3	4
1. Corbett National Park	—	0.072	0.080	0.125
2. Dudhwa National Park	0.013	—	0.092	0.173
3. Kaziranga National Park	0.012	0.013	—	0.110
4. Keibul Lamjao National Park	0.024	0.027	0.018	—
5. Southeast Asia	0.025	0.028	0.019	0.005

### Phylogenetic relationships among populations

**Figure 2:**
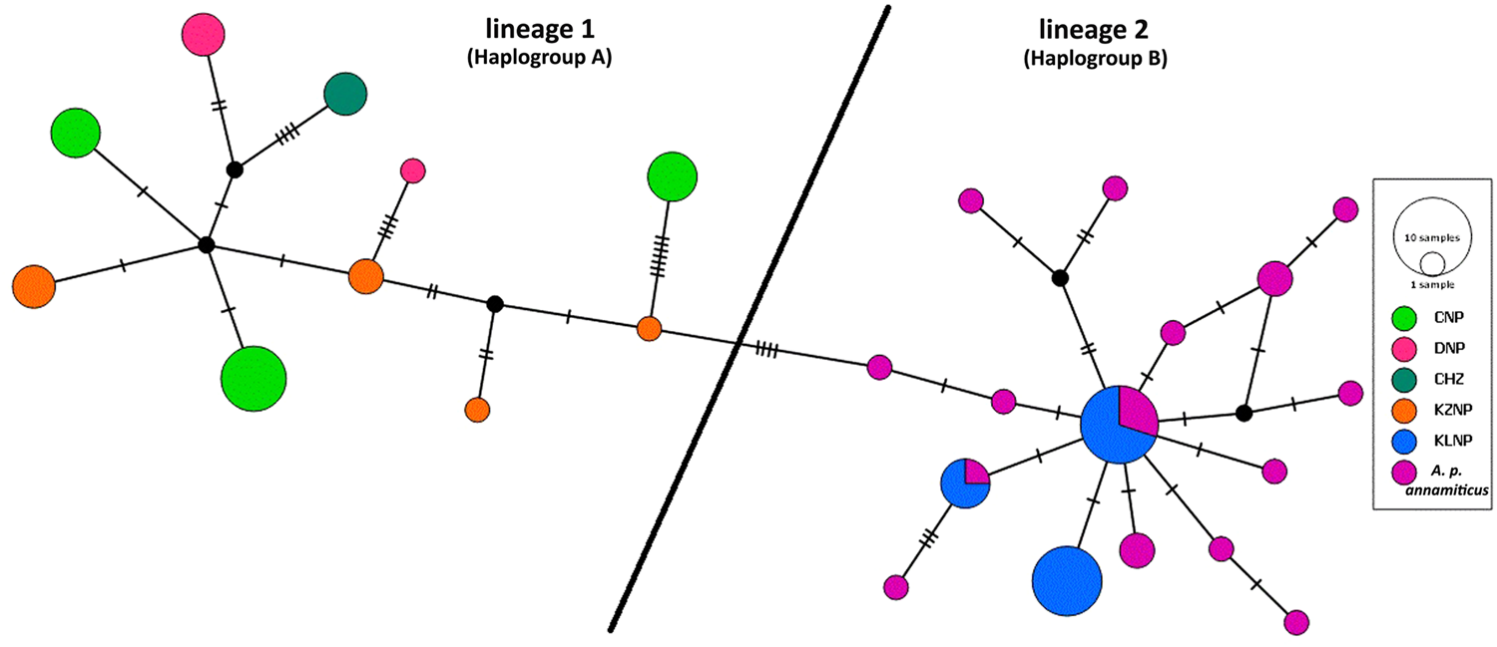
Mitochondrial DNA control region haplotype based median-joining network of hog deer populations identified two genetically distinct lineages in India. Lineage 1 consists of *A. p. porcinus* (western races) and lineage 2 consists of the subspecies *A. p. annamiticus* (eastern races) of the hog deer.

The Bayesian consensus tree showed that the mitochondrial haplotypes clustered into two main lineages. The gray shaded section (lineage 1) consists of the major haplotypes of the western race hog deer (DZ, CNP, DNP, CHZ and KZNP), and lineage 2 consists of the eastern race hog deer and the KLNP population (Supplementary Figure S1). Interestingly, all the 19 sequences submitted from Southeast Asia showed proximity to the KLNP haplotypes and clustered into lineage 2. Of these 19 sequences, 12 were derived from known *A. p. annamiticus*. The median-joining network of all the haplotypes from different locations (India and Southeast Asia) could be classified into lineage 1 and lineage 2. All the haplotypes of India except those of KLNP clustered in lineage 1 (haplogroup A), and the haplotypes of KLNP clustered along with the sequences of *A. p. annamiticus* in lineage 2 (haplogroups B) (Fig. 2).

### Divergence dating, population demographic history and neutrality test

After the alignment and mapping of all the overlapping fragments of the 16145 bp of the complete mitogenome of the hog deer from five contemporary samples (MH443786 to MH443790), we found an ~4% sequence variation between KLNP and the rest of the Indian populations of the hog deer.

**Figure 3:**
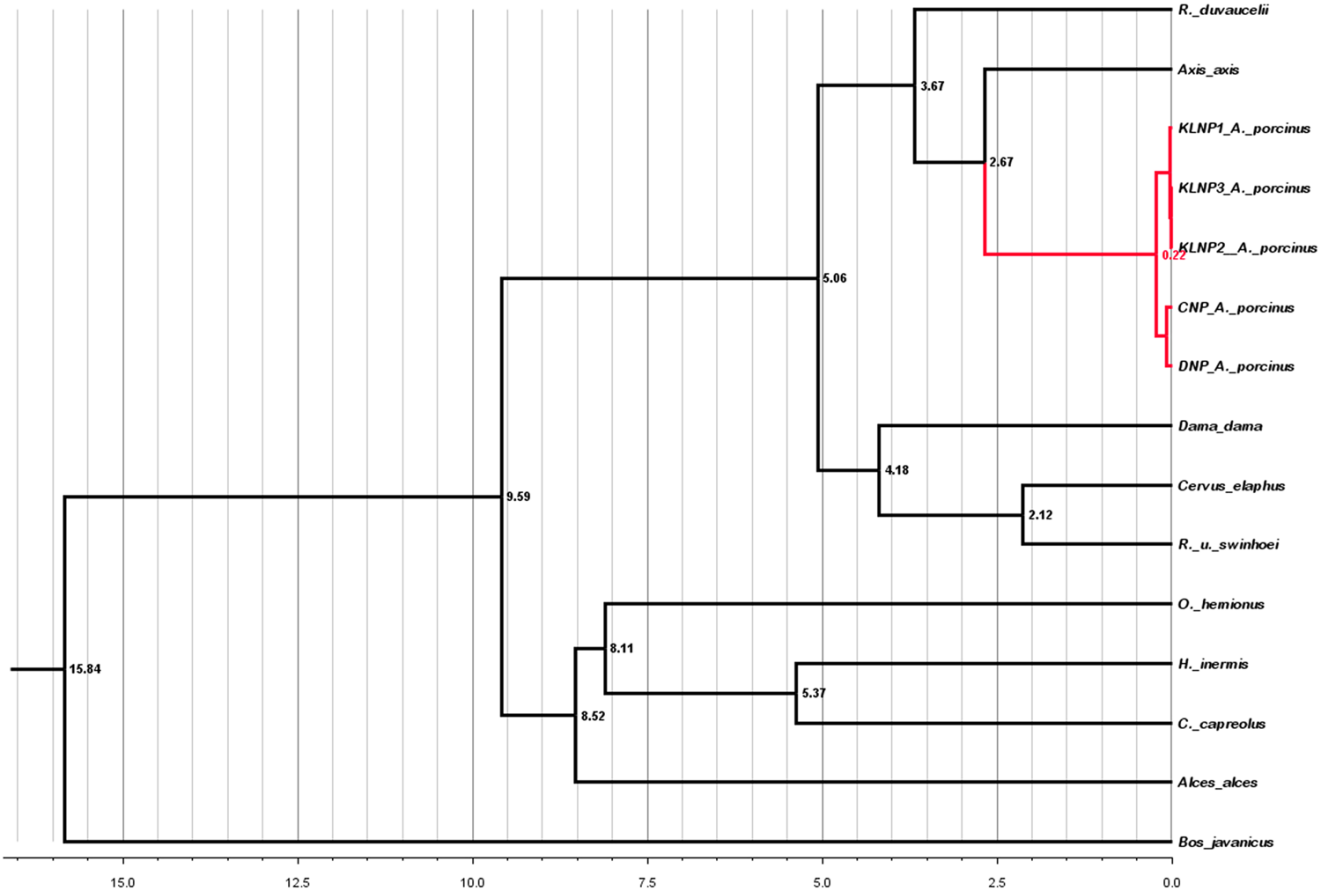
Complete mitogenome based maximum credibility tree was generated using BEAST. Molecular clocks were computed after calibrating the root age between bovids and cervids at 16.6 ± 2 Mya (95% high posterior density [HPD] = 11.7–19.8). The red clade shows that the split between the two populations of the hog deer occurred at around 0.22 Mya (95% HPD = 0.11–0.38).

The dataset was used to estimate the divergence time to the most recent common ancestor (TMRCA) of the bovids and cervids at 16.6 ± 2 Mya (95% high posterior density [HPD] = 11.7–19.8). On this basis, we estimated that the split between the *Axis* species occurred in the Late Pliocene, around 2.6 Mya (95% HPD = 1.5–4.1). Within *Axis porcinus*, the split between the western lineage (CNP and DNP) and the eastern lineage (KLNP) was estimated to have occurred at around 0.22 Mya (95% HPD = 0.11–0.38) (Fig. 3).

**Figure 4:**
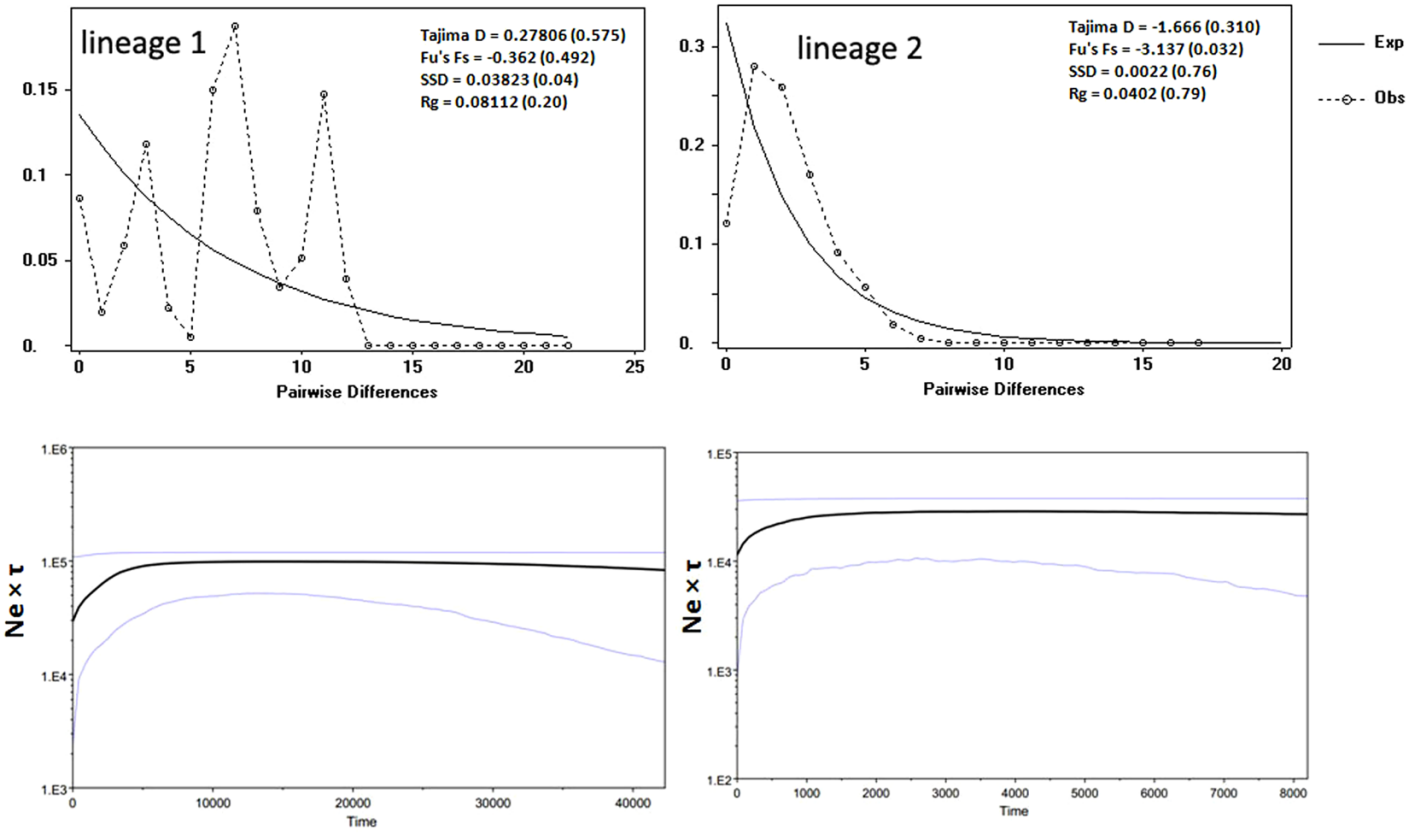
Mismatch distribution and Bayesian skyline plot (BSP) analysis of the two lineages of the hog deer using mtDNA control region gene sequence. For the mismatch distributions, the solid line shows the expected frequency, while the dashes show the observed frequency distribution. For the skyline plots, the x axis is the timing of events, assuming a substitution rate of 0.118 × 10^−6^ substitutions/site/year^54–56^. The y axis is the expressed population size, estimated as Ne × τ (Ne = effective population size; τ = generation time), and the blue lines around the solid median estimates show the 95% highest posterior density estimate of the historic effective population size.

The Bayesian skyline plots (BSPs) simulated the fluctuation of the populations over time. After a long phase of demographic stability and relatively constant population size over the last 8000–40,000 years, both the lineages of the hog deer appeared to experience a pronounced decline in effective population size over the period from ~800 years BP to 5000 years BP (Fig. 4). The demographical dynamics of the two geographical groups were inferred from the mismatch distributions. The results of Tajima’s D, Fu’s Fs tests, and raggedness statistic of mismatch distribution analyses are provided in Fig. 4. No statistical significance values for Tajima’s D and Fu’s F_S_ were observed in both the lineages of hog deer. Fu and li’s d and f tests also indicated no significant departure from neutrality (P > 0.10). The multimodal and ragged-shaped graphs in lineage 1 suggest strong population subdivision and a stable population size, which are indications of a demographic equilibrium, whereas the mismatch distribution plot for the eastern lineage was smooth and unimodal, indicating a population expansion. To assess the fitment of our data, we calculated the SSD and raggedness statistic under the demographic expansion model for each population. However, these values were not statistically significant, which indicates that neither the selective neutrality test nor the mismatch distribution test supported the hypothesis that the populations of the hog deer have passed through population expansions. Thus, the demographic pattern estimated in the BSPs and the findings of the mismatch distribution curve support each other.

### Genetic diversity

The genetic diversities of the different hog deer populations were calculated using seven microsatellite loci (Table 2). The selected microsatellite loci were highly polymorphic and exhibited a wide range of polymorphic information content (PIC), from a low value of 0.346 (Cervid1) to a high value of 0.889 (INRA001), with a mean value of 0.743. The number of alleles per locus varied from 4 (Cervid) to 13 (INRA001), with a mean of 8.710. The measures of genetic diversity were higher in DNP, CNP, and KZNP (lineage 1) than in KLNP (lineage 2). The mean allelic richness (Ar) values were higher and comparable in CNP (4.31) and DNP (4.14), whereas the value was low in the KLNP population (3.03). The observed heterozygosity (Ho) in the populations ranged between 0.491, for KLNP, and 0.615, for CNP, with a mean of 0.542 ± 0.067. The expected heterozygosity (H_E_) ranged between 0.54, for KLNP, and 0.76, for CNP, with a mean of 0.755 ± 0.072. Thus, the population of KLNP showed low genetic diversity compared with the populations of CNP, DNP, and KZNP (Table 2). The inbreeding coefficient (F_*IS*_) estimates across all populations ranged from 0.126 to 0.226.

**Table 2:** Summary of genetic diversity in hog deer populations of India.

Loci	All populations (n = 54)							Lineage 1 DNP to KZNP(n = 32)			Lineage 2 KLNP (n = 22)		
	Size Range	Na	Ne	Ho	H_E_	PIC	HWE	Na	Ho	H_E_	Na	Ho	H_E_
BM4208	151–179	11	8.480	0.720	0.879	0.871	***	10	0.719	0.862	6	0.722	0.739
INRA001	193–225	13	9.790	0.755	0.900	0.889	***	12	0.710	0.878	8	0.833	0.792
AY302223	195–223	9	4.140	0.510	0.744	0.728	***	9	0.484	0.746	3	0.556	0.619
AF232760	156–172	9	5.810	0.652	0.823	0.807	***	9	0.567	0.815	5	0.813	0.734
Cervid	164–174	4	1.590	0.250	0.340	0.346	***	4	0.387	0.479	1	0.000	0.000
RT1	205–231	10	5.570	0.440	0.816	0.801	***	10	0.625	0.849	2	0.111	0.278
T193	165–189	7	4.760	0.468	0.787	0.759	***	7	0.500	0.797	4	0.400	0.638
Mean		9	5.636	0.542	0.756	**0.743**		**8.5**	0.570	0.775	**4.143**	**0.491**	**0.543**
S.E		1.09	1.055	0.068	0.072			0.96	0.046	0.052	0.911	0.126	0.111

Key: Na = number of alleles; Ne = number of effective alleles; Ho = observed heterozygosity; H_E_ = expected heterozygosity; PIC = Polymorphic information content. HWE ***P < 0.001; Indicative adjusted nominal level (5%) = 0.001.

### Population genetic structure and genetic differentiation

**Figure 5:**
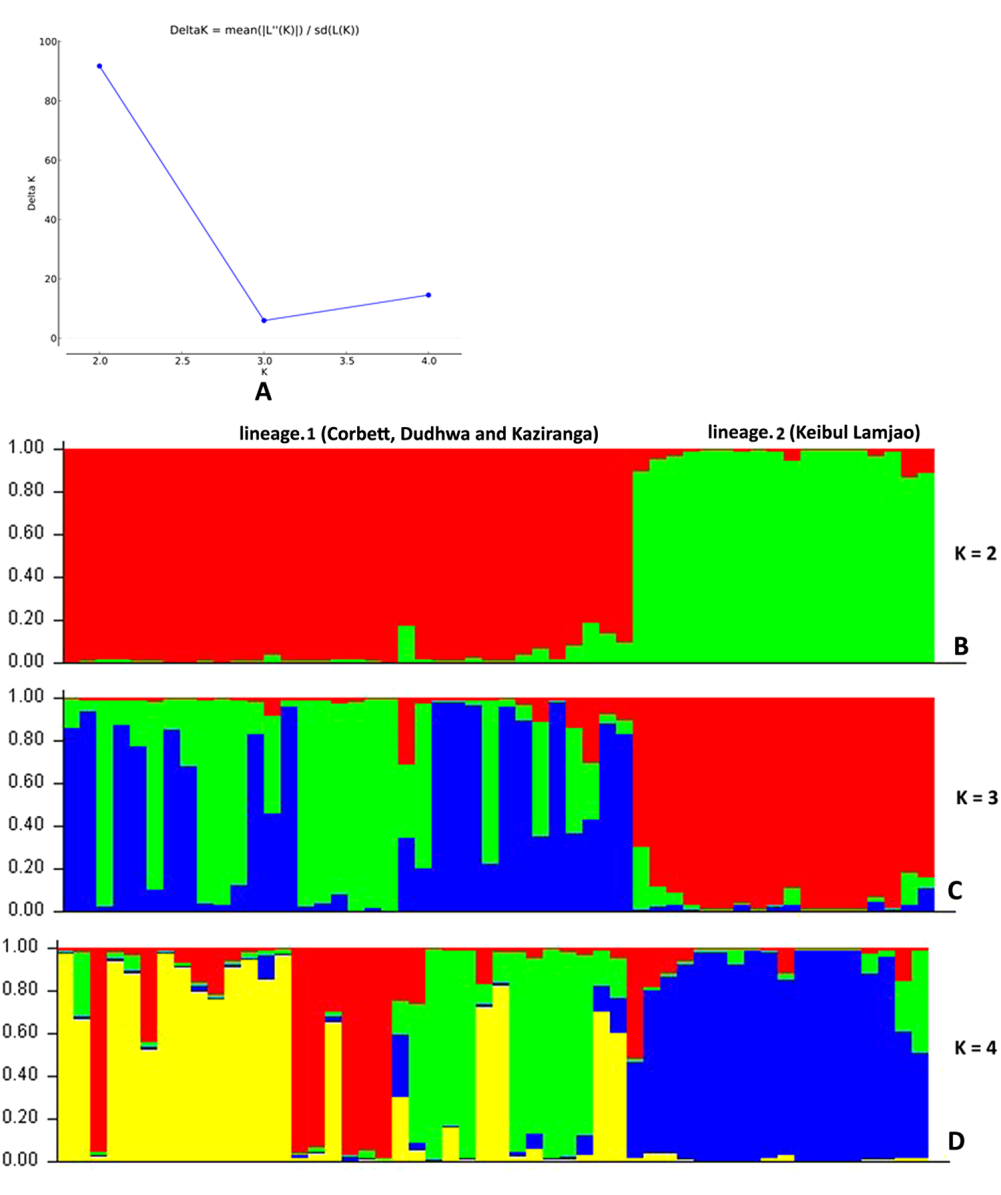
Results of microsatellite marker based Bayesian clustering structure of hog deer populations. (**A**) L(K) (mean ± SD) over four runs for each K-value; (**B**) bar plot at K = 2; (**C**) bar plots at K = 3; and (**D**) bar plots at K = 4 for population structure estimates of assignment probabilities for two lineages of the hog deer.

**Figure 6:**
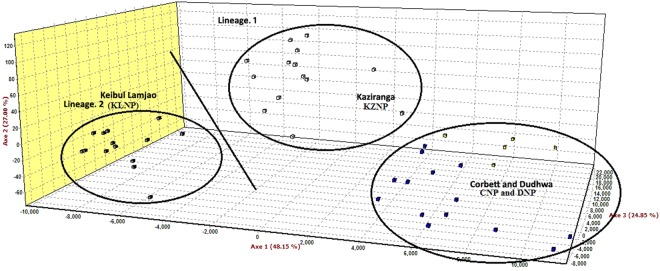
Results of factor correlation analysis (FCA) using microsatellite markers, indicating unambiguous differentiation of the hog deer populations.

**Figure 7:**
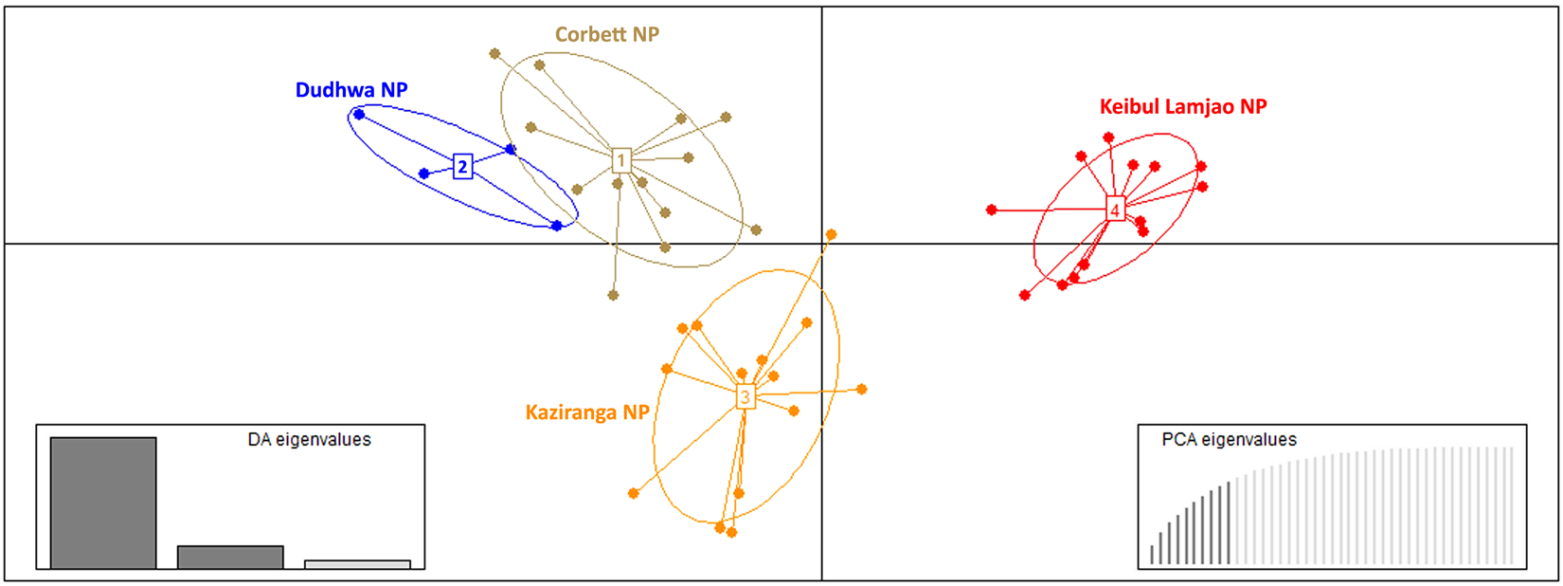
Microsatellite analysis based scatterplots of DAPC for four populations of hog deer carried out using hierarchical islands model shown by different colors and inertia ellipses, with dots representing individuals. The bar graph insets indicate the amount of variance explained by the discriminant eigen values used for plotting and represent 67% of the variance.

The Bayesian clustering analysis under the admixture model identified a high ∆K (mean likelihood of K (mean LnP [X/K] = −1263.550) value at K = 2 (Fig. 5). The two identified lineages were categorized as lineage 1, of DNP, CNP, and KZNP and lineage 2, of KLNP, which is isolated and restricted to KLNP. To obtain insights into the dataset, we also used factor correlation analysis (FCA). The sampling plot in the FCA clearly differentiated lineage 1 and lineage 2 and supported the Bayesian model-based clustering analysis (Fig. 6). This FCA plot was similar to the result of discriminant analysis of principal components (DAPC), which is congruent with the mtDNA analysis and showed that the individuals of lineage 1 are more closely related whereas the individuals of lineage 2 are distinct (Fig. 7).

The analysis based on pairwise *F*_*ST*_ for genetic differentiation of hog deer populations demonstrated significant genetic differentiation between lineage 1, of DNP, CNP, and KZNP (0.072–0.092) and lineage 2, of KLNP (>0.110) (Table 2). As with the findings from the mitochondrial region, the pairwise *F*_*ST*_ values obtained using nuclear markers were lower among the DNP, CNP, and KZNP populations than that of KLNP. This established that there was restricted gene flow between the two major groups of hog deer.

**Figure 8:**
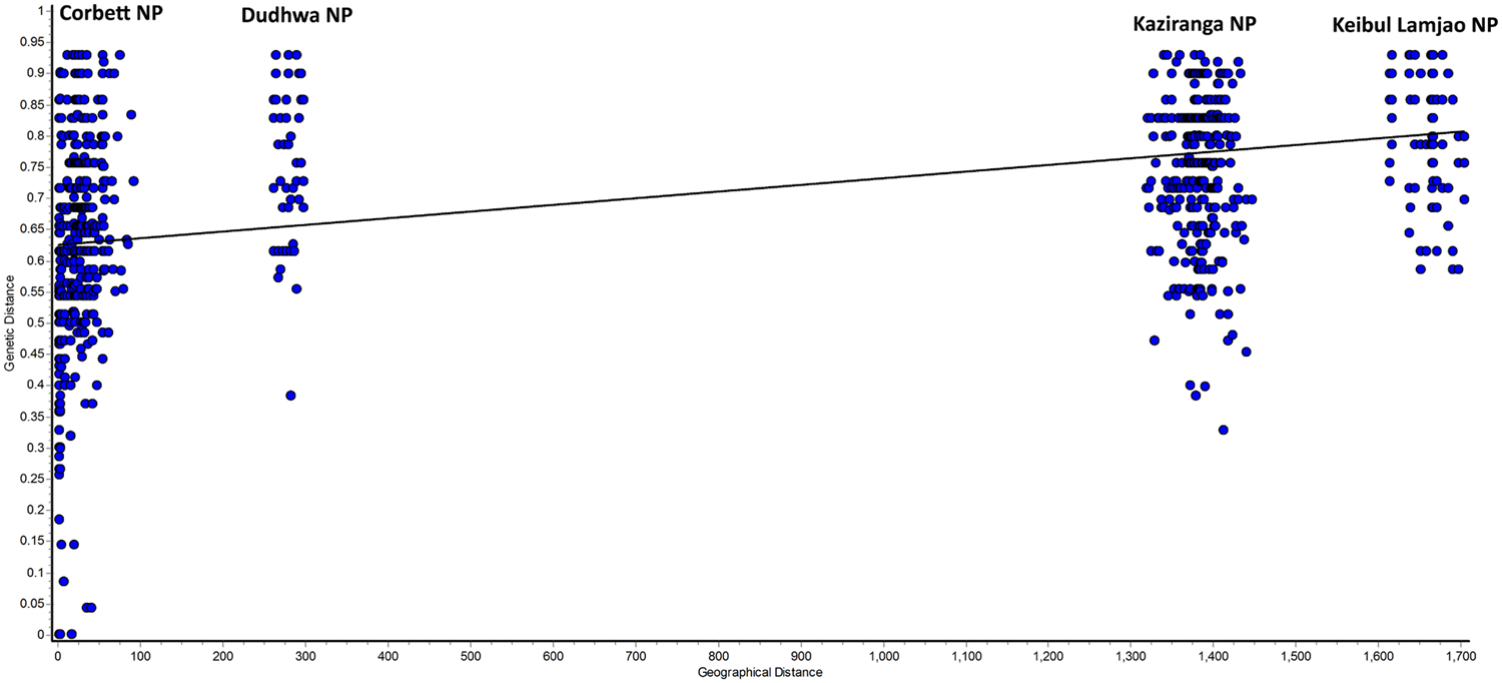
Correlation of genetic and geographical distance in kilometer between hog deer populations using microsatellite data (Mantel test, *r*_*M*_ = 0.438; *P* = 0.0009).

The spatial genetic analysis detected a significant correlation between the pairwise genetic and geographical distances for the entire study area (Mantel test, *r*_*M*_ = 0.438; *P* = 0.0009, Fig. 8). However, this pattern of the isolation by distance (IBD) was strongly influenced by the genetic differentiation (see pairwise *F*_*ST*_) and the major geographical distance between the populations of the hog deer.

## Discussion

Obtaining knowledge about the population units of a species is an important and fundamental step for guiding conservation and management authorities in wildlife management practices^27^. Several authors have reported that there are two subspecies of the hog deer^4^. The eastern race, which occurs from Myanmar eastward up to Vietnam, is known to be slightly larger than the typical western race, with distinct external features^28^. The coat color of the eastern race is brighter, more ochery, less gray, and buffy and devoid of speckling. The two forms were believed to be two separate species^28^. To assess the divergence time, genetic differentiation, phylogeographic pattern, and population genetic structure of the hog deer populations, we used both mtDNA and microsatellite markers. This study demonstrated the presence of two distinct lineages of hog deer in India. The population of KLNP showed a distinct genetic structure in comparison with major Indian populations and genetically resembled the Southeast Asian populations of *A. p. annamiticus*.

### Genetic diversity and population structure of hog deer

The pattern of genetic diversity varies with the geographic distribution of the hog deer in India. The major Indian populations of the hog deer (DNP, CNP, and KZNP) generally exhibited a high level of genetic diversity, with a large number of haplotypes diversity and numbers of alleles. In contrast, a relatively low level of genetic diversity was observed in the KLNP population. The comparatively high levels of genetic diversity in the major Indian populations might be due to the historical gene flow, whereas the KLNP population has been losing its potential connectivity with the source population of the hog deer after the glacial periods. The phylogenetic relationship and network revealed the presence of two mtDNA lineages of the hog deer in India. Interestingly, the sequences of the control region (Hap1 to 10) were found in the major Indian populations, whereas the sequence of KLNP (Hap 11 to 13) exhibited a pattern similar to that of *A. p. annamiticus*, indicating the possibility of a contemporary migration history from Southeast Asia^29^. The major Indian populations and the KLNP population can be referred to as western and eastern units. The western unit occurs from Punjab eastward through the Himalayan foothills bordering Gangetic plains up to Assam. The eastern unit extends from Manipur (KLNP), through Myanmar and Thailand up to Cambodia. It possibly extends up to Vietnam, and this needs further investigation. The structure, DAPC, and FCA analyses efficiently identified the two major clusters or ideal populations of the hog deer. The structure analysis (K = 2), supported by the other mentioned analyses, showed that the hog deer from CNP to KZNP represent one lineage and those from KLNP are from a second lineage (Fig. 5). Besides, a comparatively distinct proportion of the sequence and allelic diversity in the KLNP population support its classification as a subspecies.

### Genetic differentiation and demography

The genetic differentiation among the hog deer populations was studied using mtDNA and microsatellite markers in the current work. Significant genetic divergence between the major Indian populations of hog deer (DNP, CNP, and KZNP) and the KLNP (Manipur) population was found. Several methods were used in our analyses to obtain evidence for the genetic differentiation. The complete mitogenome was sequenced from individuals of the major Indian and KLNP populations. A sequence variation of around 4% was found, and therefore these sequences were used to estimate the divergence time along with that of other cervid and bovids species to detect the TMRCA. The major population of the hog deer in India split from the KLNP population around 0.22 Mya, and our data provide the first divergence time evidence. This divergence estimate is supported by previous molecular studies and found that the genus *Axis* separated from the *Cervus* clade during the Late Pliocene^23,30^. During the Late Pliocene, numerous species split due to the various climatic and geographical changes that acted as a driver of speciation in Southeast Asia, e.g., *Muntiacus*^31^; the Asian golden cat and the bay cat^32^; *C. perrieri* and *C. cusanus*^33^; and *Macaca* species^34^. The Bayesian skyline plot analysis strongly indicates that the hog deer population has been demographically stable over the last 8000–40,000 years and also provides evidence of a slight reduction in the effective population size over the last ~800–5000 years BP (Fig. 4).

A study on the vegetation and climate in the Eastern Himalaya showed that around 20,000 years BP the eastern parts were fully covered with open grassland and with mixed forest. Due to warm and moist climatic conditions that prevailed between 18,000 and 12,000 years BP, the forest cover was replaced with grassland^35^. Deterioration of the forest cover due to the new settlement of humans in Southeast Asia during the late Holocene might be one of the major factors for the reduction in the effective population size of the hog deer. The genus *Axis* generally occurs in a mixture of forest and open grassland^36^, and such vegetation may have extended the foothills of the Himalaya to Southeast Asia during the early Pleistocene. However, the dynamic influences of the monsoon, climatic variation, and vegetation changes during the Late Pleistocene and early Holocene might have affected the demography and population structure of the hog deer.

The limited gene flow in the KLNP region could be an indication of genetic drift due to the restricted nature of the habitat in a geographically isolated area. Interestingly, the genetic distance between the KLNP population and the recognized *A. p. annamiticus* of Southeast Asia was very low. The main factor for the separation of KLNP from the rest of the Indian population could be geographical barriers, in the form of the major Purvanchal (Northeast) mountain ridges. The Purvanchal ranges consist of the Garo, Khasi, Jalatia, Naga, Barail, and Mizo hills and might be acting as a geographical barrier between these two subspecies (*A. p. porcinus* and *A. p. annamiticus*). This theory can be well supported by specialized habitat uniqueness and isolation by distance using mantel test analysis on hog deer. Thus, the present genetic features of the KLNP population are the consequence of long-term geographical isolation and adaptation to the local environment. However, this assumption needs to be supported by additional ecological and genetic studies.

This study confirmed the existence of a small population of hog deer in Manipur that genetically resembles *A. p. annamiticus*. It indicated that the western limit of *A. p. annamiticus* is Manipur and not central Thailand as suspected earlier^4^. Similarly, KLNP (Manipur) is also known for the last remaining population of Eld’s deer (*Rucervus eldii eldii*) in eastern India, whereas the other two subspecies of Eld’s deer (*R. e. thamin* and *R. e. siamensis*) are distributed in a few localities of Indo-China and southern China, and hence this area is an ecological hotspot for similar cryptic populations. Therefore, on the basis of our mtDNA and nuclear data, we recommend that both the distinct lineages of Indian hog deer should be managed as evolutionarily significant units (ESUs). The Convention on International Trade in Endangered Species of Wild Fauna and Flora (CITES) has listed one subspecies of hog deer (*A. p. porcinus*) in Appendix III, whereas it has listed the other subspecies (*A. p. annamiticus*) in Appendix I. In a recent assessment of *A. p. annamiticus* in Southeast Asia, it was indicated that an IUCN criteria-based evaluation would justify its place in the Critically Endangered category of the IUCN Red List under criterion C1^17^. Recently, the status of the Indian hog deer was also upgraded from Schedule III to Schedule I in the Indian Wildlife (Protection) Act, 1972, which forbids hunting by more stringent rules. As the hog deer has lost ground in other countries^4^, and KLNP holds about 100 adult individuals^37^, this genetically distinct and evolutionarily significant population becomes important for the conservation of this subspecies (*A. p. annamiticus*). Considering the low population size and low genetic diversity compared with other hog deer populations and recognizing the need to conservation attention, as for the Cambodian population, it would be appropriate to upgrade the status of the hog deer (*A. p. annamiticus*) of KLNP, Manipur from Appendix III to I under CITES. The findings of this study can also be used to differentiate the two subspecies at the genetic level for management and wildlife forensics practices.

## Methods

### Sample collection and DNA extraction

We collected 77 hog deer samples comprising shed antlers, fresh fecal pellets, and tissues from dead animals (Table 3). We preserved the tissue and fecal samples in absolute ethanol and stored them at −20 °C until DNA extraction. Antlers samples were cut into small pieces and stored at room temperature. We extracted genomic DNA (gDNA) from the tissue samples using the phenol–chloroform^38^ method and from the antlers and fecal pellets using the Gu-HCl-based silica-binding method^39^. The complete mitogenome of five hog deer individuals (one each from CNP and DNP and three from KLNP) was amplified and sequenced. The samples were selected on the basis of the quality of the gDNA. The samples were collected from the remains of dead animals or non-invasively. Hence, the approval of the animal ethical committee was not required for the experiments. The samples were obtained from the field with the permission of the Chief Wildlife Warden and Park Manager of the state and field. We confirm that all the experiments were performed in accordance with the relevant guidelines and regulations.

**Table 3:** Details of samples used for the analysis of genetic variations of hog deer from India.

Origin	Status	Sample code	mtDNA		Microsatellite	
			n	Types	n	Types
Dudhwa National Park, Uttar Pradesh, India	wild	DNP	4	A(4)	4	A(4)
Corbett National Park, Uttarakhand, India	wild	CNP	14	A(6); F(8)	14	A(6); F(8)
Chattbir zoo Chandigarh, India	captive	CHZ	3	A(3)	—	—
Kaziranga National Park, Assam, India	wild	KZNP	8	A(8)	14	A(14)
Keibul Lamjao National Park, Manipur, India	wild	KLNP	48	T(18);F(30)	22	T(18),F(4)

### PCR amplification of mtDNA region and sequencing

PCR amplification was carried out in 20-μl volumes containing 10–20 ng of the extracted genomic DNA, 1 × PCR buffer (Applied Biosystems), 2.0 mM MgCl_2_, 0.2 mM of each dNTP, 3 pmol of each primer, and 0.5 units of AmpliTaq Gold DNA polymerase (Applied Biosystems). We performed PCR amplification using 23 overlapping fragments of the complete mtDNA^40^ and variable portions of the control region primers, Cerv.tPro, and CervCRH^41^. The thermal cycling conditions for both the primer pair was as follows: an initial hot start at 95 °C of 10 minutes, followed by 35 cycles at 95 °C for 45 seconds, 55 °C for 45 seconds and 72 °C for 1 minute, with a final extension of 72 °C for 15 minutes. The efficiency and reliability of the PCR reactions were monitored using control reactions. The PCR products were electrophoresed on 2% agarose gel and visualized under UV light in the presence of ethidium bromide dye. The amplified PCR products were treated with Exonuclease-I and Shrimp Alkaline Phosphatase (USB, Cleveland, OH) for 15 minutes each at 37 °C and 80 °C, respectively, to remove any residual primer. The purified fragments were sequenced directly in an Applied Biosystems Genetic Analyzer 3500XL from both primers set using a BigDye v3.1 Kit.

### PCR amplification of microsatellite loci and genotyping

Seven microsatellite loci (BM4208, INRA001, AY302223, AF232760, Cervid1, RT1, and T193) were used for genetic analysis of the hog deer^42–48^. Multiplex amplifications were carried out in 10-μl reaction volumes containing 5 μl of QIAGEN Multiplex PCR Buffer Mix (QIAGEN Inc.), 0.2 μM labeled forward primer (Applied Biosystems), 0.2 μM unlabeled reverse primer, and 20–100 ng of the template DNA. PCR was performed under the following conditions: initial denaturation at 95 °C for 15 min, followed by 35 cycles of 95 °C for 45 seconds, 55 °C for 1 minute and 72 °C for 1 minute, with a final extension of 60 °C for 30 minutes. Alleles were resolved in an ABI 3500XL Genetic Analyzer (Applied Biosystems) using the LIZ 500 Size Standard (Applied Biosystems) and analyzed using GeneMapper version 3.7 (Applied Biosystems).

## Data Analysis

### Genetic diversity, phylogenetic and demographic analysis

In addition to 77 sequences of hog deer generated in-house, weobtained 19 hog deer sequences from GenBank (NCBI). Twelve of these sequences had been generated from the eastern subspecies, *A. p. annamiticus*. Sequences derived from the forward and reverse directions were aligned and edited using SEQUENCHER® version 4.9 (Gene Codes Corporation, Ann Arbor, MI, USA). These sequences were aligned separately using the CLUSTAL X 1.8 multiple alignment program^49^, and the alignments were checked by visual inspection. DnaSP 5.0^50^ was used to analyze the haplotype diversity (h), nucleotide diversity (p), and polymorphic sites (s). The numbers of nucleotide substitutions per site were estimated for multiple substitutions using the Tamura-3 parameter method in MEGA7^51^. Insertion–deletion (INDEL) sites were excluded from the sequence analysis. For the genetic distance, we used the Tamura-3 parameter using the discrete Gamma distribution (TN92 + G) with lowest BIC score value. Phylogenetic analyses were conducted in BEAST ver 1.7^52^. One sequence of *Elaphurus davidianus* (accession number AF291894) was used as an out-group for better insights into the phylogenetic relationships. The spatial distribution of the haplotypes was visualized using a median-joining network, which was created using the PopART software package^53^.

A Bayesian skyline plot (BSP) was constructed using the Monte Carlo Markov Chain (MCMC) method using BEAST ver 1.7^52^. The temporal trends in the effective population size of the hog deer over time/generations were estimated using a coalescent BSP. We used HKY and empirical base frequencies models under a strict molecular clock and a stepwise skyline model with a substitution rate of 0.118 × 10^–6^ substitutions/site/year^54–56^.

Mismatch distribution graphs were plotted using DnaSP 5.0^50^ to infer if the hog deer had experienced a demographic expansion, equilibrium, or bottleneck. Different neutrality statistical approaches were used to investigate the demographic history of each sample and each population. Tajima’s D^57^, Fu’s Fs^58^ and Fu and Li’s F and D^59^ are appropriate statistics for short DNA sequences and were used to test whether the sequences conformed to the expectations of neutrality using DnaSP 5.0^50^. In general, the significant negative value of Tajima’s D and Fu’s Fs test is an indication of population demographic expansion. Besides, the goodness-of-fit of distribution with expected distribution using a model of population expansion by estimating the sum of squared deviations (SSD) and Harpending’s raggedness index (Rg) by assessing significance with 1000 bootstrap replicates were calculated to test for demographic changes in the hog deer populations using Arlequin version 3.5.1.2^60^.

### Estimating divergence dating

The complete mitochondrial genomes of five individuals were sequenced (MH443786 to MH443790), and the sequences were used to date the divergence of the hog deer populations. The complete mtDNA sequence of *A. porcinus* (JN632600) was used as a reference to map the generated sequences. To estimate the divergence times of the hog deer populations, we inferred genealogies using a relaxed lognormal clock model in BEAST v.1.7^52^. We performed the analysis using 10 sequences downloaded from the NCBI database, including the sequences of nine species of the family Cervidae, i.e., the swamp deer (*R. duvaucelii*, NC020743), sambar (*R. unicolor*, NC008414), chital (*A. axis*, JN632599), fallow deer (*D. dama*, JN632629), red deer (*C. elaphus*, AB245427), Eurasian elk (*A. alces*, JN632595), European roe deer (*C. capreolus*, KJ681491), water deer (*H. inermis*, NC011821), and mule deer (*O. hemionus*, JN632670) and the sequence of one species of the family Bovidae, i.e., the banteng (*B. javanicus*, FJ997262). The TMRCA of bovids and cervids was set as a calibration point to 16.6 ± 2 million years ago (Mya) with a normal prior distribution^31,61^. We used a Yule-type speciation model and the HKY + I + G substitution rate model for tree reconstruction. We subsequently used the TMRCA estimate of the *Cervus*-*Axis* split as the tree prior for the calibrations within our dataset. We conducted two independent analyses, using MCMC lengths of 10 million generations, logging every 1000 generation. All the runs were evaluated in Tracer v. 1.6. The final phylogenetic tree was visualized in FigTree v.1.4.2. (http://tree.bio.ed.ac.uk/software/figtree/).

### Microsatellite analysis

Based on the amplification success of the microsatellite loci, we selected 54 hog deer samples for microsatellite analysis (Table 2). The polymorphic information content (PIC), the number of alleles per locus, and the observed (Ho) and expected (HE) heterozygosity were estimated using the program CERVUS, version 3.0.6^62^. The linkage disequilibrium was tested using the statistical software package GENEPOP 3.4^63^. The allelic richness (Ar), mean inbreeding coefficient (FIS)^64^, and pairwise FST values (gene flow) among the populations were estimated using FSTAT, version 2.9.3^65^. We checked all the loci at the population level for deviation from the Hardy–Weinberg equilibrium (HWE) using an exact test in GENEPOP 3.4^63^. CONVERT 1.31^66^ was used to change the input file into the required formats. The existence of population genetic structure in hog deer, the number of genetic clusters (K) to all individuals were estimated by model-based Bayesian assignment method using Structure 2.3.2^67^. In this method, independent assessments are made for each cluster without prior information about the origin of the population. The log-likelihood data [Ln Pr (X/K)] were estimated for the given K between 1 and 10 with ten independent runs set by 5,00,000 Markov chain Monte Carlo (MCMC) iterations, followed by a burn-in period of 50,000 iterations. The highest hierarchical level of Delta K was determined by comparing the log-likelihood [Ln Pr (X/K)] estimates at different values of K using Structure Harvester^68^. Populations were separated by factor correlation analysis (FCA) using the GENETIX 4.05 software package^69^.

### Genetic differentiation

We carried out assignment and clustering of the populations using the DAPC method and the ADEGENET package in R^70^. DAPC is a multivariate approach that maximizes the genetic differentiation between groups with unknown prior clusters, thus improving the discrimination of populations. The pairwise *F*_*ST*_ values (gene flow) among the populations were estimated using FSTAT, version 2.9.3^65^.

### Spatial genetic analysis

Alleles in Space 1.0^71^ was used to correlate the pairwise genetic and geographic distances by detecting the pattern of IBD between the disjointed areas with hog deer according to Mantel’s test.

## Electronic supplementary material

Supplementary Tables and Figures

## Data Availability

The sequence data are available through NCBI GenBank.
