# Supplementary material for: Genetic analysis of endangered hog deer (Axis porcinus) reveals two distinct lineages from the Indian subcontinent
Source: Sci Rep. 2018 Nov 5;8:16308. doi: 10.1038/s41598-018-34482-9 (PMC6218551; doi:10.1038/s41598-018-34482-9)
Supplement: Supplementary file 1 — Supplementary Tables and Figures [file 41598_2018_34482_MOESM1_ESM.doc]

**Supplementary materials**

**Genetic analysis of endangered hog deer (*Axis porcinus*)** **reveals two distinct lineages from the Indian subcontinent**

**Sandeep Kumar Gupta^1^*^#^, Ajit Kumar^1#^, Sangeeta Angom^1^, Bhim Singh^1^, Mirza Ghazanfar Ullah Ghazi^1^, Chongpi Tuboi^1^, Syed Ainul Hussain^1^***

1. Wildlife Institute of India, P.O. Box # 18, Chandrabani, Dehra Dun-248001, Uttarakhand, India

# Equal contribution

****Corresponding author(s)***

| Dr. S. K. Gupta | Dr. S. A. Hussain |  |
| --- | --- | --- |
| Scientist E | Scientist G |  |
| Wildlife Institute of India, | Wildlife Institute of India, |  |
| Chandrabani, Dehra Dun  248 001 (U.K.), India  E-mail: skg@wii.gov.in, | Chandrabani, Dehra Dun  248 001 (U.K.), India  E-mail: hussain@wii.gov.in, |  |
| Telephone: +91-135-2646343 | Telephone: +91-135-2646210 |  |
| Fax No: +91-135-2640117 | Fax No: +91-135-2640117 |  |

**Running head: Phylogeography of hog deer, *Axis porcinus***

**Supplementary Table ST1:** Details of the mtDNA control region sequences used in this study.

| **Haplotypes** | **Sample Size** | **Subspecies** | **Accession Number/Sample origin** | **Reference** |
| --- | --- | --- | --- | --- |
| HD1 | 1 | *A. p. porcinus* | EU870592 (India) | Bhaskar & Goyal (Unpublished) |
|  | 6 | *A. p. porcinus* | Corbett National Park, UK, India (MH392156) | This study |
| HD2 | 4 | *A. p. porcinus* | Corbett National Park, UK, India (MH392157) | This study |
| HD3 | 4 | *A. p. porcinus* | Corbett National Park, UK, India (MH392158) | This study |
| HD4 | 1 | *A. p. porcinus* | Dudwa National Park, UP, India (MH392159) | This study |
| HD5 | 3 | *A. p. porcinus* | Dudwa National Park, UP, India (MH392160) | This study |
| HD6 | 3 | *A. p. porcinus* | ChattBir zoo Chandigarh, India (MH392161) | This study |
| HD7 | 1 | *A. p. porcinus* | Kaziranga National Park, Assam, India (MH392162) | This study |
| HD8 | 2 | *A. p. porcinus* | Kaziranga National Park, Assam, India (MH392163) | This study |
| HD9 | 3 | *A. p. porcinus* | Kaziranga National Park, Assam, India (MH392164) | This study |
| HD10 | 2 | *A. p. porcinus* | Kaziranga National Park, Assam, India (MH392165) | This study |
| HD11 | 8 | *A. p. porcinus* | Keibul Lamjao National Park, Manipur, India (MH392166) | This study |
| HD12 | 7 | *A. p. porcinus* | Keibul Lamjao National Park, Manipur, India (MH392167) | This study |
|  | 1 | *A. porcinus* | EF491201 (Thailand) | Wajjwalku et al. (Unpublished) |
|  | 1 | *A. p. annamiticus* | KM881619 (Thailand) | Wajjwalku et al. (Unpublished) |
|  | 1 | *A. p. annamiticus* | KM881624 (Thailand) | Wajjwalku et al. (Unpublished) |
| HD13 | 3 | *A. p. porcinus* | Keibul Lamjao National Park, Manipur, India (MH392168) | This study |
|  | 1 | *A. p. annamiticus* | KM881622 (Thailand) | Wajjwalku et al. (Unpublished) |
| HD14 | 1 | *A. porcinus* | EF491198 (Thailand) | Wajjwalku et al. (Unpublished) |
| HD15 | 1 | *A. porcinus* | EF491199 (Thailand) | Wajjwalku et al. (Unpublished) |
|  | 1 | *A. p. annamiticus* | KM881616 (Thailand) | Wajjwalku et al. (Unpublished) |
| HD16 | 1 | *A. porcinus* | EF491200 (Thailand) | Wajjwalku et al. (Unpublished) |
| HD17 | 1 | *A. porcinus* | EF491202 (Thailand) | Wajjwalku et al. (Unpublished) |
|  | 1 | *A. p. annamiticus* | KM881623 (Thailand) | Wajjwalku et al. (Unpublished) |
| HD18 | 1 | *A. porcinus* | EF491203 (Thailand) | Wajjwalku et al. (Unpublished) |
| HD19 | 1 | *A. porcinus* | EF491205 (Thailand) | Wajjwalku et al. (Unpublished) |
| HD20 | 1 | *A. p. annamiticus* | KM881614 (Thailand) | Wajjwalku et al. (Unpublished) |
| HD21 | 1 | *A. p. annamiticus* | KM881615 (Thailand) | Wajjwalku et al. (Unpublished) |
| HD22 | 1 | *A. p. annamiticus* | KM881617 (Thailand) | Wajjwalku et al. (Unpublished) |
| HD23 | 1 | *A. p. annamiticus* | KM881618 (Thailand) | Wajjwalku et al. (Unpublished) |
| HD24 | 1 | *A. p. annamiticus* | KM881620 (Thailand) | Wajjwalku et al. (Unpublished) |
| HD25 | 1 | *A. p. annamiticus* | KM881621 (Thailand) | Wajjwalku et al. (Unpublished) |
| HD26 | 1 | *A. p. annamiticus* | KM881625 (Thailand) | Wajjwalku et al. (Unpublished) |

**Supplementary Table ST2.** Polymorphic sites showing 26 haplotypes of mtDNA control region of hog deer. Dots (.) denote identity with the first sequence. Numerics in the top three rows represent the polymorphic positions. n represents the number of samples; N is the IUPAC code represent any base at polymorphic positions.

|  |  |  | **1** | **1** | **1** | **1** | **1** | **1** | **1** | **1** | **2** | **2** | **2** | **2** | **2** | **2** | **2** | **2** | **2** | **2** | **2** | **2** | **2** | **3** | **3** | **3** | **3** | **3** | **3** | **3** | **3** | **3** | **3** | **3** | **4** | **4** | **4** | **4** | **4** |
| --- | --- | --- | --- | --- | --- | --- | --- | --- | --- | --- | --- | --- | --- | --- | --- | --- | --- | --- | --- | --- | --- | --- | --- | --- | --- | --- | --- | --- | --- | --- | --- | --- | --- | --- | --- | --- | --- | --- | --- |
|  |  | **5** | **0** | **1** | **1** | **1** | **2** | **3** | **3** | **4** | **0** | **0** | **1** | **4** | **4** | **6** | **6** | **7** | **7** | **7** | **8** | **8** | **9** | **0** | **0** | **0** | **1** | **2** | **3** | **5** | **7** | **8** | **8** | **8** | **1** | **1** | **1** | **1** | **4** |
| **Hap** | **n** | **2** | **2** | **1** | **4** | **6** | **2** | **4** | **5** | **9** | **3** | **9** | **4** | **2** | **3** | **1** | **9** | **0** | **1** | **6** | **0** | **7** | **5** | **4** | **6** | **8** | **1** | **2** | **6** | **1** | **2** | **1** | **2** | **7** | **5** | **6** | **8** | **9** | **2** |
| **HD1** | 7 | T | T | T | A | C | T | A | C | C | A | C | T | T | C | A | A | T | A | T | C | A | A | C | A | C | T | G | A | T | A | T | A | G | G | T | C | T | C |
| **HD2** | 4 | . | . | . | . | . | C | . | . | . | G | . | . | C | . | . | G | . | G | . | . | . | . | T | G | . | C | . | . | C | . | . | G | . | A | . | . | . | . |
| **HD3** | 4 | . | . | . | . | . | C | . | . | . | . | . | . | . | . | . | . | . | . | . | . | . | . | . | . | T | . | . | . | . | . | . | . | . | . | . | . | . | . |
| **HD4** | 1 | C | . | . | . | . | C | . | . | . | . | . | . | C | . | . | G | . | . | . | T | . | . | . | G | . | . | . | . | . | . | . | . | . | A | . | . | . | . |
| **HD5** | 3 | . | . | . | . | . | C | . | . | . | G | . | C | . | . | . | . | . | . | . | . | . | . | . | . | . | . | . | . | . | . | . | G | . | A | C | . | C | . |
| **HD6** | 3 | . | . | C | . | . | C | . | . | T | G | . | . | . | . | . | . | . | . | . | . | . | G | . | . | . | C | . | . | . | . | . | . | . | . | . | . | . | . |
| **HD7** | 1 | . | . | . | . | . | C | . | . | . | G | . | . | C | T | . | . | C | . | . | . | . | . | . | . | . | . | . | . | C | . | . | . | . | A | . | . | . | . |
| **HD8** | 2 | . | . | . | . | . | C | . | . | . | . | . | . | C | . | . | . | . | . | . | . | . | . | . | . | . | . | . | . | . | . | . | . | . | A | . | . | . | . |
| **HD9** | 3 | . | . | . | . | . | C | . | . | . | . | . | . | . | T | . | . | . | . | . | . | . | . | . | . | . | . | . | . | . | . | . | . | . | . | C | . | . | . |
| **HD10** | 1 | . | . | . | . | . | C | . | . | . | . | . | . | C | . | . | . | C | . | . | . | . | . | . | G | . | . | . | . | C | . | . | . | . | A | . | . | . | . |
| **HD11** | 8 | . | . | . | . | T | C | . | . | . | . | . | . | C | T | . | . | . | . | C | . | G | G | . | G | . | . | A | . | C | . | . | . | . | A | . | . | . | T |
| **HD12** | 10 | . | . | . | . | T | C | . | . | . | . | . | . | C | T | . | . | . | . | C | . | G | G | . | G | . | . | . | . | C | . | . | . | . | A | . | T | . | T |
| **HD13** | 4 | . | . | . | . | T | C | . | . | . | . | . | . | C | . | . | . | . | . | C | . | G | G | . | G | . | . | . | . | C | . | . | . | . | A | . | T | . | T |
| **HD14** | 1 | N | . | . | . | T | C | . | . | . | . | . | . | C | T | G | . | . | . | C | . | G | G | . | G | . | . | . | G | C | . | . | . | . | N | N | N | N | N |
| **HD15** | 2 | N | . | . | . | T | C | . | . | . | . | . | . | C | T | . | . | . | . | . | . | G | G | . | G | . | . | . | . | C | . | . | . | . | A | . | N | N | N |
| **HD16** | 1 | N | C | . | C | T | C | C | A | . | . | . | . | C | T | . | . | . | . | C | . | G | G | . | G | . | . | . | . | C | . | . | . | . | N | N | N | N | N |
| **HD17** | 2 | N | . | . | . | T | C | . | . | . | . | . | . | C | T | . | . | . | . | C | . | G | G | . | G | T | . | . | . | C | . | . | . | A | N | N | N | N | N |
| **HD18** | 1 | N | . | . | C | T | C | C | . | . | . | T | . | C | T | . | . | . | . | C | . | G | G | . | G | . | . | . | . | C | . | . | . | . | N | N | N | N | N |
| **HD19** | 1 | N | . | . | . | T | C | . | . | . | . | . | . | C | T | . | . | . | . | C | . | G | G | . | G | T | . | . | . | C | . | A | . | . | A | . | N | N | N |
| **HD20** | 1 | N | . | . | . | T | C | . | . | . | . | . | . | C | T | . | . | . | . | C | . | G | G | . | G | . | . | . | . | C | . | . | . | A | N | N | N | N | N |
| **HD21** | 1 | N | . | . | . | T | C | . | . | . | . | . | . | C | T | G | . | . | . | C | . | G | G | . | G | . | . | . | . | C | . | . | . | . | N | N | N | N | N |
| **HD22** | 1 | N | . | . | . | T | C | . | . | . | . | . | . | C | T | . | . | . | . | C | . | . | G | . | G | T | . | . | . | C | . | . | . | A | N | N | N | N | N |
| **HD23** | 1 | N | . | . | . | T | C | . | . | . | . | . | . | C | T | . | . | C | . | C | . | . | G | . | G | . | . | . | . | C | . | . | . | . | N | N | N | N | N |
| **HD24** | 1 | N | . | . | . | T | C | . | . | . | . | . | . | C | T | . | G | . | . | C | . | G | G | . | G | . | . | . | . | C | . | . | . | . | N | N | N | N | N |
| **HD25** | 1 | N | . | . | . | T | C | . | . | . | . | . | . | C | T | . | . | . | . | C | . | . | G | . | G | . | . | . | . | C | . | . | . | . | N | N | N | N | N |
| **HD26** | 1 | N | . | . | . | T | C | . | . | . | . | . | . | . | . | . | . | . | . | C | . | G | G | . | G | . | . | . | . | C | G | . | . | . | N | N | N | N | N |

**Supplementary Table ST3.** Number of variable sites (V) and Parsimony informative sites (Pi) in the conserved and coding region genes across the complete mitogenomes of 5 individuals of hog deer (*Axis porcinus*). Positions site were identified by comparing with *Axis porcinus* (JN632600).

| **mtDNA region** | **12s** | **16s** | **ND1** | **ND2** | **Cox1** | **Cox2** | **Cox3** | **ND3** | **ND4L** | **ND4** | **ND5** | **ND6** | **Cytb** | **D-loop** |
| --- | --- | --- | --- | --- | --- | --- | --- | --- | --- | --- | --- | --- | --- | --- |
| Position site | 70-1025 | 1093-2662 | 2740-3695 | 3905-4946 | 5330-6874 | 7018-7701 | 8615-9398 | 9468-9813 | 9884-10180 | 10174-11551 | 11752-13572 | 13556-14083 | 14157-15296 | 15442-16145 |
| V | 2 | 2 | 2 | 5 | 11 | 1 | 2 | 1 | 0 | 4 | 9 | 3 | 6 | 15 |
| Pi | 1 | 2 | 2 | 3 | 11 | 1 | 2 | 1 | 0 | 4 | 7 | 3 | 4 | 14 |


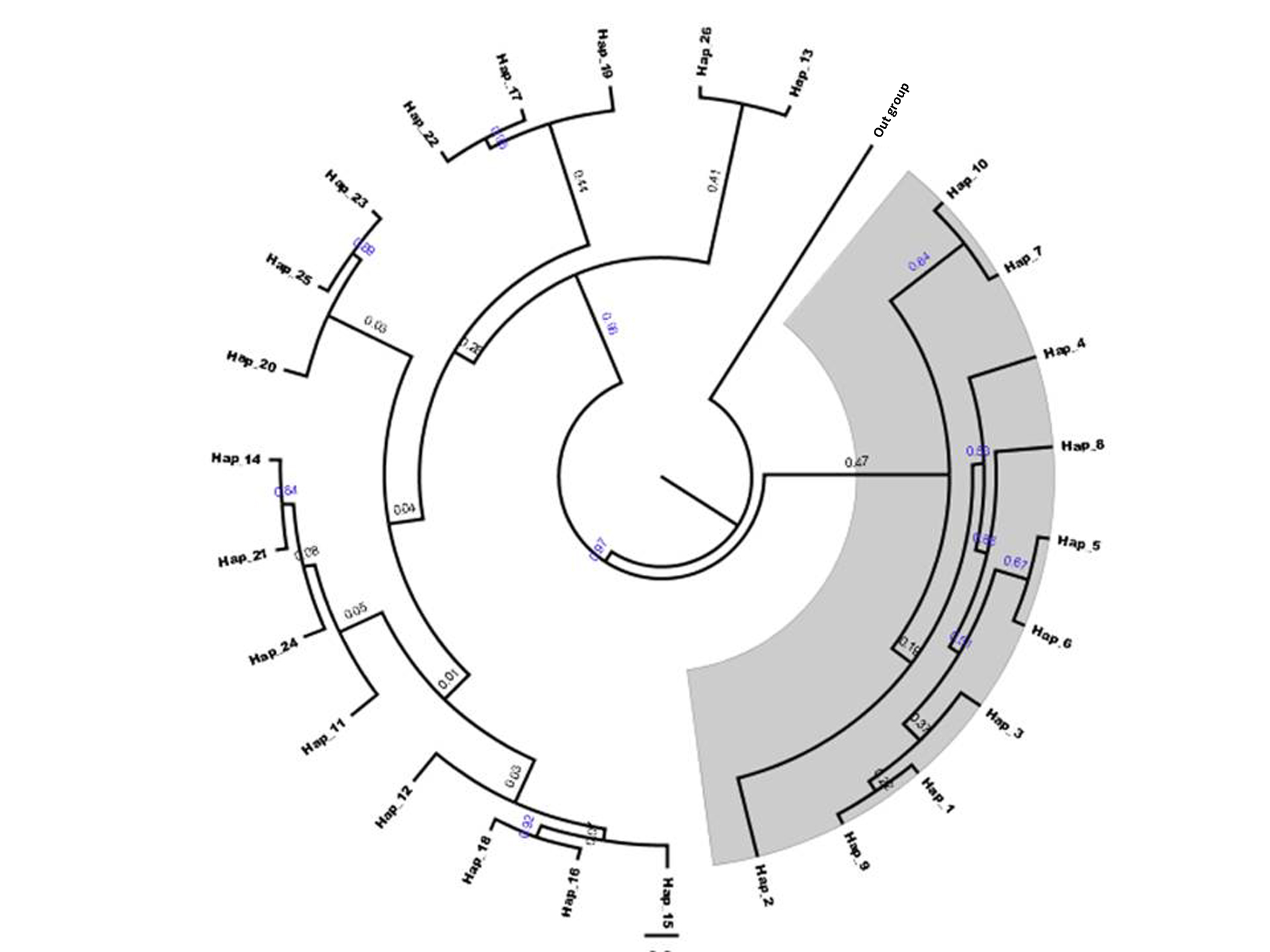


**Supplementary Figure S1.** Mitochondrial control region based Bayesian (MCMC) consensus tree for 26 hog deer haplotypes. Posterior values are given at their respective nodes. The *Elaphurus davidianus* (AF291894) was used as outgroup. Gray shade represents clade of *A. p. porcinus* (lineage 1) and branches without shade exhibits *A. p. annamiticus* (lineage 2) of hog deer.


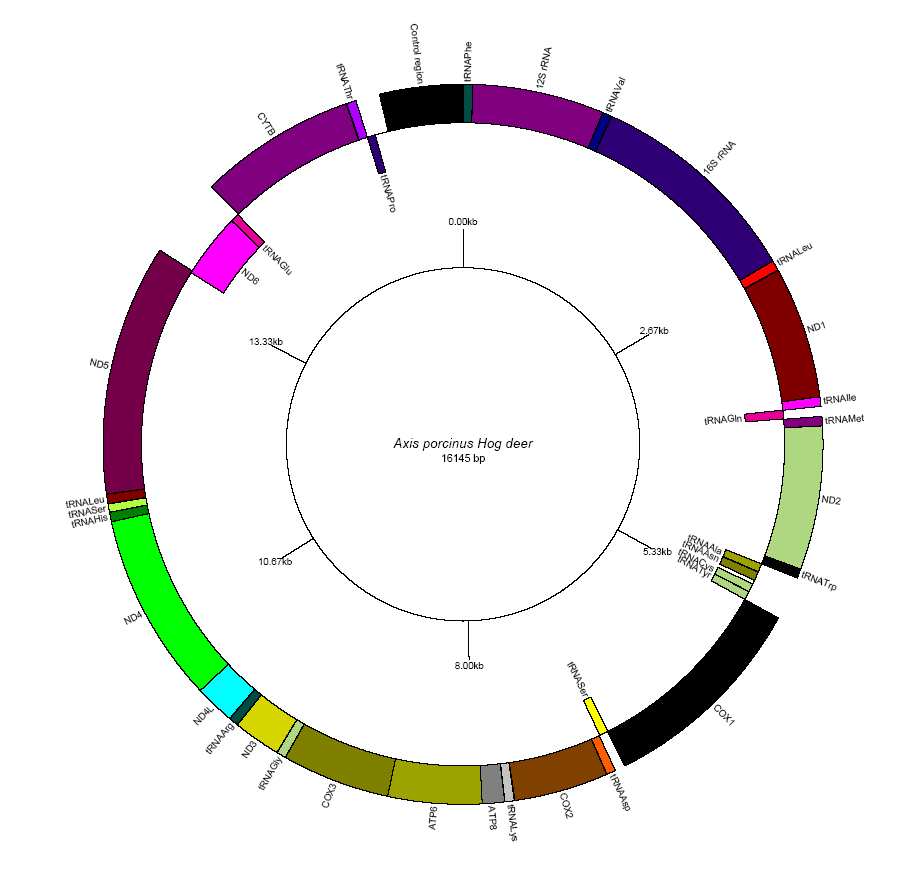


**Supplementary Figure S2.** Mitogenome of *A. p. porcinus* and *A. p. annamiticus* drawn from 5 complete mtDNA sequence (MH443786 - MH443790) using GenomeVx software.
